# Neuroprotective effects of bavachalcone in a mouse model of Parkinson’s disease: linking the gut-brain axis and systemic metabolism

**DOI:** 10.3389/fnins.2025.1650288

**Published:** 2025-09-22

**Authors:** Shuai Shi, Xiaohan Bian, Mengyun Li, Xiaobing Zhang, Wenji Huang, Xian Shao, Tiantian Lu, Xuebin Yu

**Affiliations:** ^1^Department of Neurosurgery, Shaoxing People’s Hospital, Shaoxing, Zhejiang, China; ^2^Department of Medical Research Center, Shaoxing People’s Hospital, Shaoxing, Zhejiang, China; ^3^Department of Endocrinology, Shaoxing People’s Hospital, Shaoxing, Zhejiang, China

**Keywords:** bavachalcone, Parkinson’s disease, neuroprotection, gut microbiota, metabolomics, MPTP

## Abstract

**Background:**

Parkinson’s disease (PD) is a progressive neurodegenerative disorder characterized by motor dysfunction and dopaminergic neuronal loss. Emerging evidence suggests that gut microbiota dysbiosis and systemic metabolic disturbances contribute to the pathogenesis of PD. This study aimed to investigate the neuroprotective effects of bavachalcone, a prenylated chalcone isolated from *Psoralea corylifolia*, in an MPTP-induced mouse model of PD, with a particular focus on its effects on motor function, inflammation, gut microbiota, and serum metabolism.

**Methods:**

Male C57BL/6 mice were divided into Control, MPTP, Bavac-L (low-dose bavachalcone), and Bavac-H (high-dose bavachalcone) groups. Bavachalcone was administered by gavage, followed by MPTP injection to induce PD. Behavioral assessments (open field test, pole test, and rotarod test), western blotting, immunohistochemistry, immunofluorescence, 16S rDNA sequencing of fecal microbiota, and untargeted metabolomics of serum were performed to evaluate the effects of bavachalcone.

**Results:**

Bavachalcone significantly alleviated MPTP-induced motor impairment, preserved dopaminergic neurons in the substantia nigra and striatum, and reduced systemic inflammation and glial activation. Gut microbiota analysis showed that bavachalcone improved microbial richness and diversity, enriched beneficial genera, such as *Allobaculum*, and suppressed harmful taxa, such as *Ligilactobacillus* and *Helicobacter*. Metabolomic profiling revealed that bavachalcone modulated pathways, including pyruvate metabolism, folate biosynthesis, and phenylalanine metabolism.

**Conclusion:**

Bavachalcone exerts neuroprotective effects in mice with PD by improving motor function, preserving dopaminergic neurons, reducing inflammation, modulating gut microbiota composition, and remodeling systemic metabolism. These findings highlight bavachalcone as a promising therapeutic candidate for PD.

## Introduction

Parkinson’s disease (PD) is the second most common neurodegenerative disorder after Alzheimer’s disease, affecting more than 6 million people worldwide, with a prevalence of approximately 1% among individuals over 60 years of age (Tysnes and Storstein, 2017). Clinically, PD is characterized by bradykinesia, resting tremor, muscular rigidity, and postural instability. Its pathological hallmark is the progressive degeneration of dopaminergic neurons in the substantia nigra pars compacta (Ramesh and Arachchige, 2023). Despite extensive research, the precise pathogenesis of PD remains incompletely understood, and current therapeutic strategies mainly provide symptomatic relief without halting disease progression.

In recent years, accumulating evidence has highlighted the important role of the gut-brain axis in the pathogenesis of PD (Houser and Tansey, 2017). The gut microbiota can influence central nervous system functions through multiple mechanisms, including the modulation of immune responses, production of microbial metabolites, and regulation of the enteric nervous system (Liu et al., 2022). Dysbiosis of the gut microbiota is commonly observed in patients with PD, suggesting that intestinal inflammation and microbiota-derived neuroactive molecules may contribute to neurodegenerative processes (Kleine Bardenhorst et al., 2023; Safarpour et al., 2022). Thus, the modulation of the gut microbiota has been proposed as a promising therapeutic strategy for PD.

Because of their broad therapeutic potential and the ease of structural modification on both the A and B rings, chalcones represent promising scaffolds for the development of novel treatments for PD and other neurodegenerative diseases (Królicka et al., 2022; Parambi et al., 2020). Bavachalcone is a naturally occurring, prenylated flavonoid isolated from the traditional Chinese medicinal plant *Psoralea corylifolia*. Bavachalcone has demonstrated protective effects in models of oxidative stress and neuroinflammation, suggesting its potential applications in the treatment of neurodegenerative diseases (Dang et al., 2015; Wu et al., 2023). Notably, bavachalcone can penetrate the blood-brain barrier and accumulate within brain tissue (Yang et al., 2018). However, there have been no systematic investigations into the role of bavachalcone in PD or its effects on the gut-brain axis.

In this study, we used an MPTP-induced mouse model of PD to systematically evaluate the effects of bavachalcone on motor dysfunction, dopaminergic neuronal survival, gut microbiota composition, and the serum metabolic profile. Our aim was to explore the neuroprotective mechanisms of bavachalcone and to provide new insights into potential therapeutic strategies for PD.

## Materials and methods

### Animals and treatment

Eight-week-old male C57BL/6 mice (23 ± 2 g) were obtained from Shanghai Model Organisms Center, Inc. The mice were maintained in a standard environment (temperature, 22 ± 2 °C; humidity, 50% ± 10%) under specific pathogen-free conditions. All animal experiments were approved by the Animal Ethics Committee of Shaoxing People’s Hospital (Approval no. 2024Z078). The mice were randomly assigned to four groups (*n* = 6 per group): (1) Control group, (2) MPTP model group (MPTP), (3) low-dose bavachalcone + MPTP group (Bavac-L, 30 mg/kg), (Aladdin, China; Cat. No. 28448–85-3), and (4) high-dose bavachalcone + MPTP group (Bavac-H, 60 mg/kg).

All animals underwent daily oral gavage for seven consecutive days. The Bavac-L and Bavac-H groups were administered bavachalcone, respectively, whereas the Control and MPTP groups received phosphate-buffered saline (PBS, pH 7.4). On day 7 post-treatment, mice in the MPTP, Bavac-L, and Bavac-H groups were intraperitoneally injected with MPTP (25 mg/kg/d, Sigma-Aldrich, USA; Cat. No. M0896) for seven consecutive days. An equivalent volume of isotonic sodium chloride solution was administered to the Control group.

All surgical procedures were performed under anesthesia induced by intraperitoneal injection of 0.3% pentobarbital sodium (50 mg/kg; Sigma-Aldrich, USA; Cat. No. 4390-16-3), with rigorous measures taken to minimize animal suffering. The study protocol was approved by the Institutional Animal Care and Use Committee of Shaoxing People’s Hospital (Shaoxing, China; ID number: 2024Z078) and strictly complied with China’s Regulations for the Administration of Affairs Concerning Experimental Animals. All experimental procedures conformed to the ARRIVE guidelines for preclinical research.

### Behavioral assessments

#### Pole test

The pole test was performed to evaluate motor coordination and bradykinesia. A vertical wooden pole (120 cm high and 3 cm in diameter) was used. During training, mice were placed at the top of the pole with their heads oriented upward and allowed to descend to the base three times for acclimatization. On the test day (24 h-72 h after the final MPTP administration), each mouse was placed head-up at the top of the pole, and the time required to descend completely to the base was recorded. Three trials were conducted per mouse at 1 h intervals, and the average descent time was calculated for analysis.

#### Rotarod test

The rotarod test was conducted to assess motor coordination and balance in mice. Mice were placed on an automated rotating rod at 30 rpm for up to 180 s. Each mouse underwent three training sessions (1 h apart). During the formal test, the total distance traveled by mice during falled form rotarod was recorded. Three trials were performed per mouse with 1 h inter-trial intervals, and the average distance was used for statistical analysis.

#### Open field test

Before testing, mice were acclimatized to the experimental environment for 30 min. Each mouse was placed in the center of a dark experimental box (25 × 25 cm), and its activity was recorded during a 3 min observation period. The total distance traveled was calculated to assess spontaneous locomotor activity.

### Western blotting analysis

Brain tissues from the striatum were rapidly isolated and stored at −80 °C. Tissues were homogenized in 1 × RIPA lysis buffer supplemented with 1 mM phenylmethylsulfonyl fluoride and a phosphatase inhibitor cocktail (Roche, Basel, Switzerland). The homogenates were centrifuged at 12,000 g for 15 min at 4 °C, and the supernatants were collected for protein analysis.

Equal amounts of total protein from each group were separated by 12% SDS-polyacrylamide gel electrophoresis and transferred onto 0.45 μm polyvinylidene difluoride membranes. Membranes were blocked with 5% (w/v) skim milk in Tris-buffered saline containing 0.1% Tween-20 for 1 h at room temperature to prevent nonspecific binding.

Subsequently, membranes were incubated overnight at 4 °C with primary antibodies, including mouse anti-tyrosine hydroxylase (TH) antibody (Sigma, USA; Cat. No. AB152; 1:1000) and mouse anti-GAPDH antibody (Proteintech, China; Cat. No. 60004-1-Ig; 1:1000). After washing, the membranes were incubated with horseradish peroxidase-conjugated goat anti-mouse secondary antibodies (Cell Signaling Technology, USA; 1:1000) for 1 h at room temperature. Protein bands were visualized using an enhanced chemiluminescence detection kit and imaged using an Invitrogen iBright 1,500 imaging system.

### Immunohistochemistry

Brain samples were collected and fixed in 4% paraformaldehyde at −4 °C for subsequent preparation of paraffin-embedded sections. Mouse brain tissue sections were deparaffinized using environmentally friendly dewaxing reagents and dehydrated using anhydrous ethanol. Sections were incubated overnight at 4 °C with a rabbit monoclonal anti-TH primary antibody (Sigma, USA; Cat. No. AB152; 1:200). Following three washes with PBS, the sections were incubated with horseradish peroxidase-conjugated goat anti-rabbit IgG secondary antibody (Proteintech, China; Cat. No. SA00001-2; 1:200) for 50 min at room temperature, then washed three times with PBS. TH immunoreactivity was visualized using DAB chromogenic substrate, and images were acquired using a light microscope.

### Immunofluorescence

Sections were deparaffinized in xylene and rehydrated using a graded ethanol series. Antigen retrieval was performed by boiling the sections in 10 mM citrate buffer (pH 6.0) for 15 min. After cooling, sections were blocked with 5% normal goat serum containing 0.3% Triton X-100 for 1 h at room temperature.

Primary antibodies against GFAP (Abcam, UK; Cat. No. ab7260; 1:1000) and IBA1 (Wako, Japan; Cat. No. 019–19,741; 1:500) were applied overnight at 4 °C. After washing with PBS, the sections were incubated with Alexa Fluor 594- or Alexa Fluor 488-conjugated secondary antibodies (Invitrogen, USA; Cat. No. 8890 & 4,412; 1:1000) for 1 h at room temperature in the dark. Nuclei were counterstained with DAPI (Proteintech, China; Cat. No. 28718-90-3; 1 μg/mL). Images were captured using a fluorescence microscope.

### Enzyme-linked immunosorbent assay (ELISA)

Whole blood samples were collected from the mice via cardiac puncture and allowed to clot at room temperature. Serum was separated by centrifugation at 3000 rpm for 10 min at 4 °C and stored at −80 °C until analysis.

Serum levels of TNF-*α*, IL-1β, and IL-6 were quantified using commercial ELISA kits (Byabscience Biotechnology Co., Ltd., China; Cat. No. BY-EM220852, BY-EM220174 and BY-EM220188) according to the manufacturer’s protocols. Absorbance was measured at 450 nm using a microplate reader, and cytokine concentrations were calculated based on standard curves.

### Gut microbiota 16S rDNA sequencing and analysis

Fresh fecal samples were collected from mice and immediately stored at −80 °C. Total bacterial genomic DNA was extracted using a commercial extraction kit according to the manufacturer’s instructions. The V3–V4 hypervariable regions of the bacterial 16S rRNA gene were amplified using PCR with specific primers. Amplicons were purified, quantified, and sequenced on an Illumina MiSeq platform.

Raw sequencing data were quality-filtered, merged, and clustered into operational taxonomic units at a 97% similarity threshold using QIIME software. Alpha diversity indices, including Shannon, Chao1, and ACE, were calculated to assess species richness and diversity. Beta diversity was analyzed using Principal Coordinate Analysis and nonmetric multidimensional scaling based on Bray–Curtis distances. Differential microbial taxa among groups were identified using LEfSe analysis, and the taxonomic composition was visualized using community bar plots at various taxonomic levels (family, genus, and species). Data analysis was performed using majorbio tools created by Major-BIO Co. Ltd. (Shanghai, China) at https://cloud.majorbio.com/.

### Untargeted serum metabolomics analysis

Untargeted metabolomic profiling was performed using liquid chromatography–tandem mass spectrometry with a high-resolution mass analyzer (Thermo Fisher Q Exactive). Chromatographic separation was achieved using a C18 reverse-phase column with gradient elution. Data acquisition was performed in both positive and negative ion modes.

Raw data were processed for peak alignment, normalization, and identification using Compound Discoverer software. Multivariate statistical analyses—including principal component analysis, partial least squares discriminant analysis (PLS-DA), and orthogonal PLS-DA—were conducted to identify global metabolic differences. Differential metabolites were selected based on a variable importance in projection value >1.0 and *p* < 0.05. KEGG pathway enrichment analysis was performed to explore the biological pathways involved.

### Statistical analysis

All statistical analyses were conducted using GraphPad Prism 9.0 (GraphPad Software, Inc., San Diego, CA, USA). At least three independent replicates were used to ensure accuracy. Statistical analyses were performed using GraphPad Prism 5. Student’s t-test was used to compare two groups, and one-way analysis of variance with Tukey’s or Bonferroni’s multiple comparison test was used to assess the significance in more than two groups. Data are presented as the mean ± standard error of the mean. Statistical differences between groups were evaluated, and *p*-values < 0.05 were considered statistically significant. Significance levels are indicated as follows: *p* < 0.05, *p < 0.01, **p < 0.001, and ***p < 0.0001.

## Results

### Bavachalcone alleviates MPTP-induced behavioral impairments in mice

**Figure 1:**
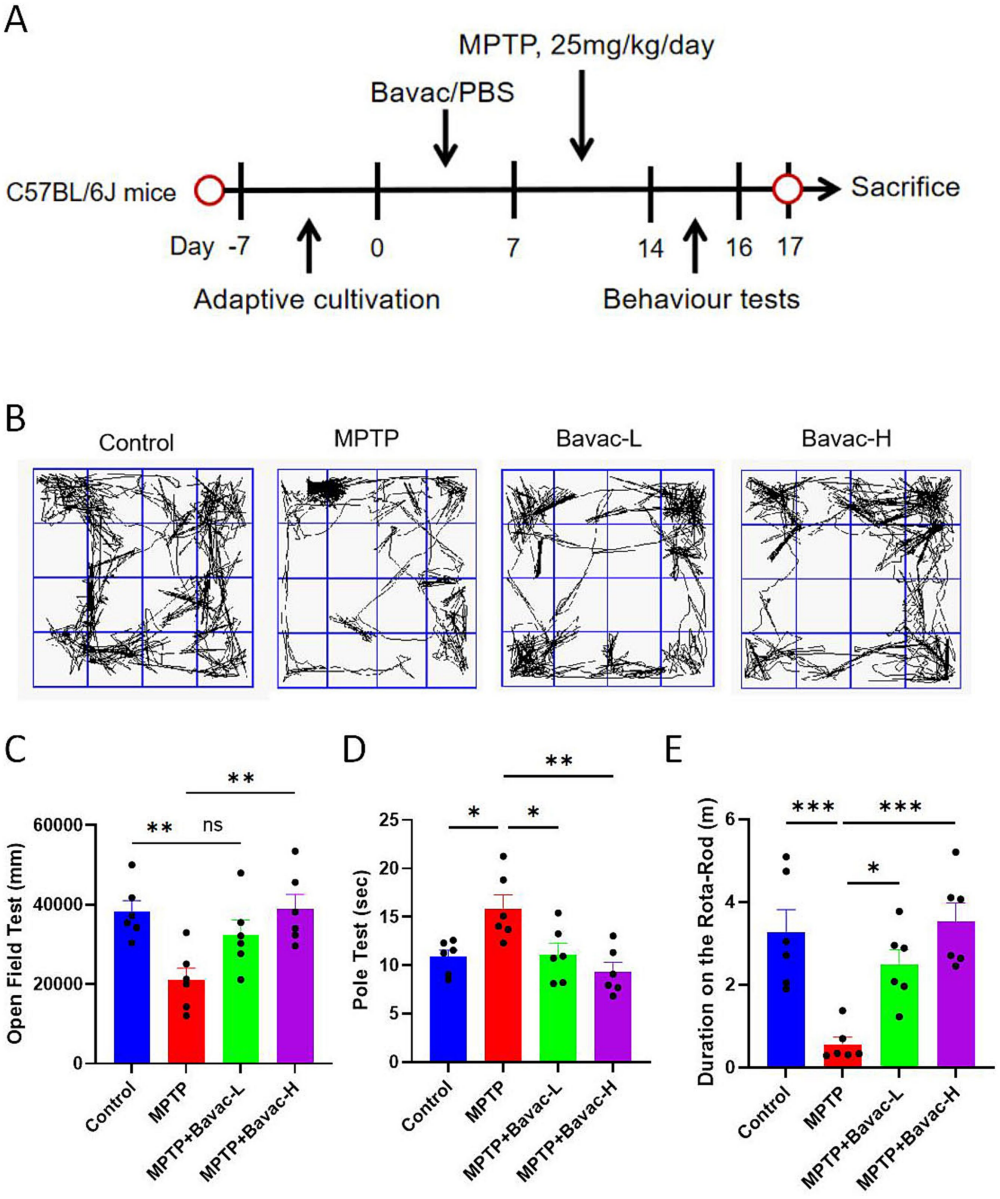
Bavachalcone ameliorates motor deficits in PD model mice. **(A)** Schematic of the animal experimental procedure. **(B)** Representative images from open field tests. **(C)** Total distance moved in the open field test. **(D)** Time to descend the pole in the pole test. **(E)** Distance to fall in the rotarod test. Data are expressed as mean ± SEM (*n* = 6 per group). *p < 0.05, **p < 0.01, ***p < 0.001. PD, Parkinson’s disease; SEM, standard error of the mean.

To investigate the protective effects of bavachalcone, a bioactive compound derived from *Psoralea corylifolia*, in a mouse model of PD, mice were randomly assigned to four groups: Control, MPTP model, Bavac-L, and Bavac-H. Bavachalcone was administered via oral gavage for seven consecutive days, followed by intraperitoneal MPTP injection to induce the Parkinsonian phenotype. After model induction, behavioral assessments—including the open field test, pole test, and rotarod test—were performed to evaluate the therapeutic effect of bavachalcone on motor deficits (Figure 1A).

The open field test was used to assess spontaneous locomotor activity, a general indicator of motor performance. Mice in the MPTP group showed a significant reduction in total distance traveled and less complex movement trajectories compared with those in the Control group, indicating impaired locomotor function. By contrast, mice treated with bavachalcone exhibited increased total distance traveled and more complex movement patterns, suggesting that bavachalcone mitigated MPTP-induced motor impairment (Figures 1B,C).

The pole and rotarod tests were used to assess bradykinesia and motor coordination, respectively. MPTP treatment significantly increased the time required to descend the pole, whereas bavachalcone treatment significantly reduced this latency (Figure 1D). In the rotarod test, mice in the MPTP-treated group exhibited significantly reduced travel distance, whereas those administered bavachalcone demonstrated markedly increased travel distance on the rotating rod, indicating improved motor coordination (Figure 1E). Collectively, these behavioral results demonstrate that bavachalcone attenuates MPTP-induced motor dysfunction in mice.

### Bavachalcone attenuates MPTP-induced dopaminergic neurodegeneration in the substantia nigra and striatum of mice with PD

**Figure 2:**
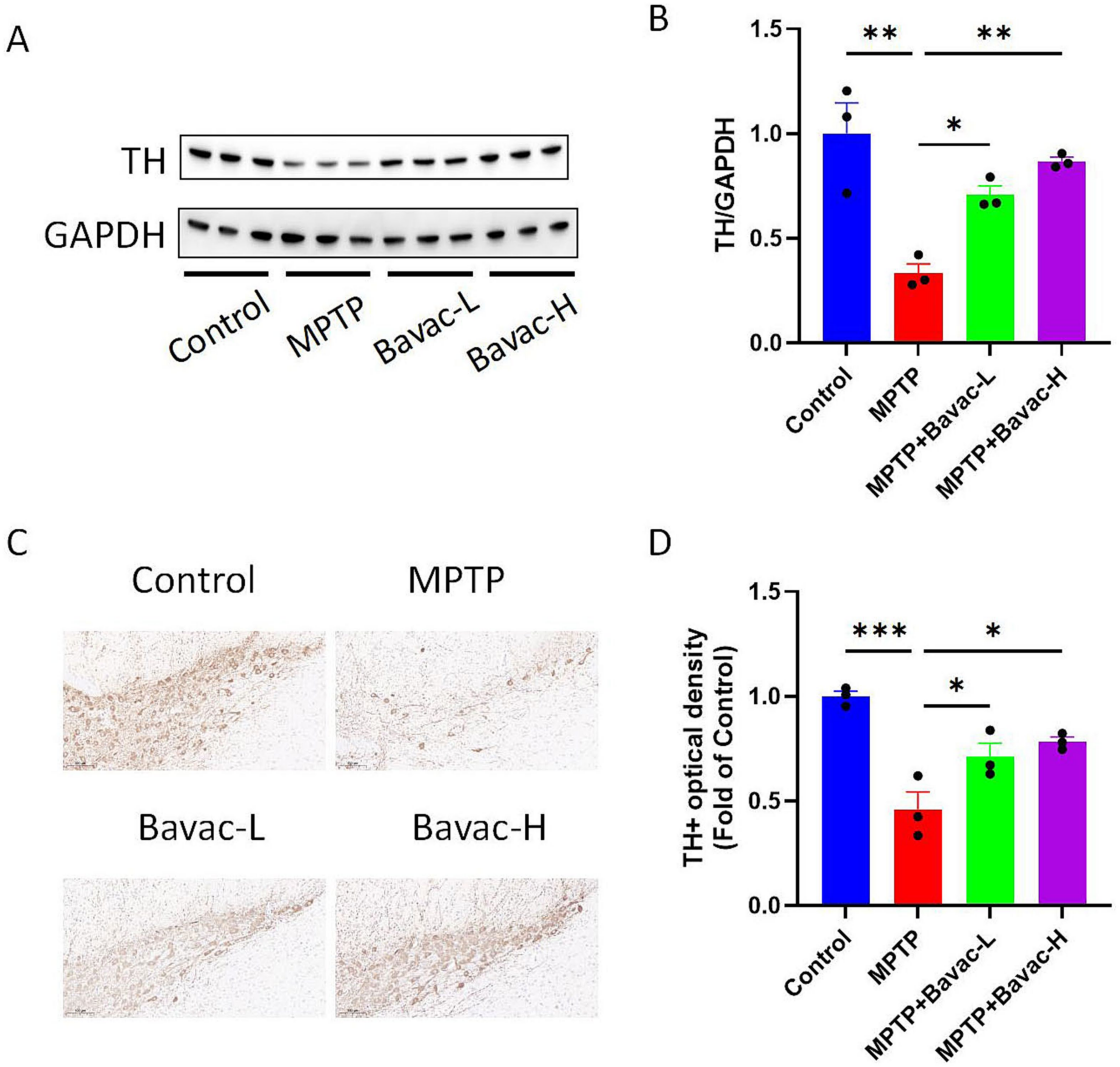
Bavachalcone restores TH expression in the striatum and substantia nigra. **(A)** Representative western blot of TH in the striatum. **(B)** Quantification of TH protein expression in the striatum. **(C)** Representative images of TH immunofluorescence staining in the substantia nigra of mice. **(D)** Quantification of TH-positive neurons in the substantia nigra. Data are expressed as mean ± SEM (*n* = 3 per group). *p < 0.05, **p < 0.01, ***p < 0.001. TH, tyrosine hydroxylase.

To evaluate dopaminergic neuronal damage, TH protein expression in the striatum was assessed using western blotting. TH levels were significantly reduced in the striatum of MPTP-treated mice compared with those in the control group. Notably, bavachalcone treatment partially restored TH expression levels relative to those in the MPTP group (Figures 2A,B), suggesting a neuroprotective effect.

Immunohistochemical staining was performed to detect TH-positive neurons in the substantia nigra (Figures 2C,D). Compared with the control group, the MPTP group showed a significant reduction in the number of TH-positive dopaminergic neurons. However, bavachalcone administration resulted in a notable increase in the number of TH-positive neurons relative to that in the MPTP group.

These findings indicate that bavachalcone effectively mitigates MPTP-induced dopaminergic neurodegeneration in the nigrostriatal pathway in the mouse model of PD.

### Bavachalcone reduces systemic inflammation and glial activation in mice with MPTP-induced PD

**Figure 3:**
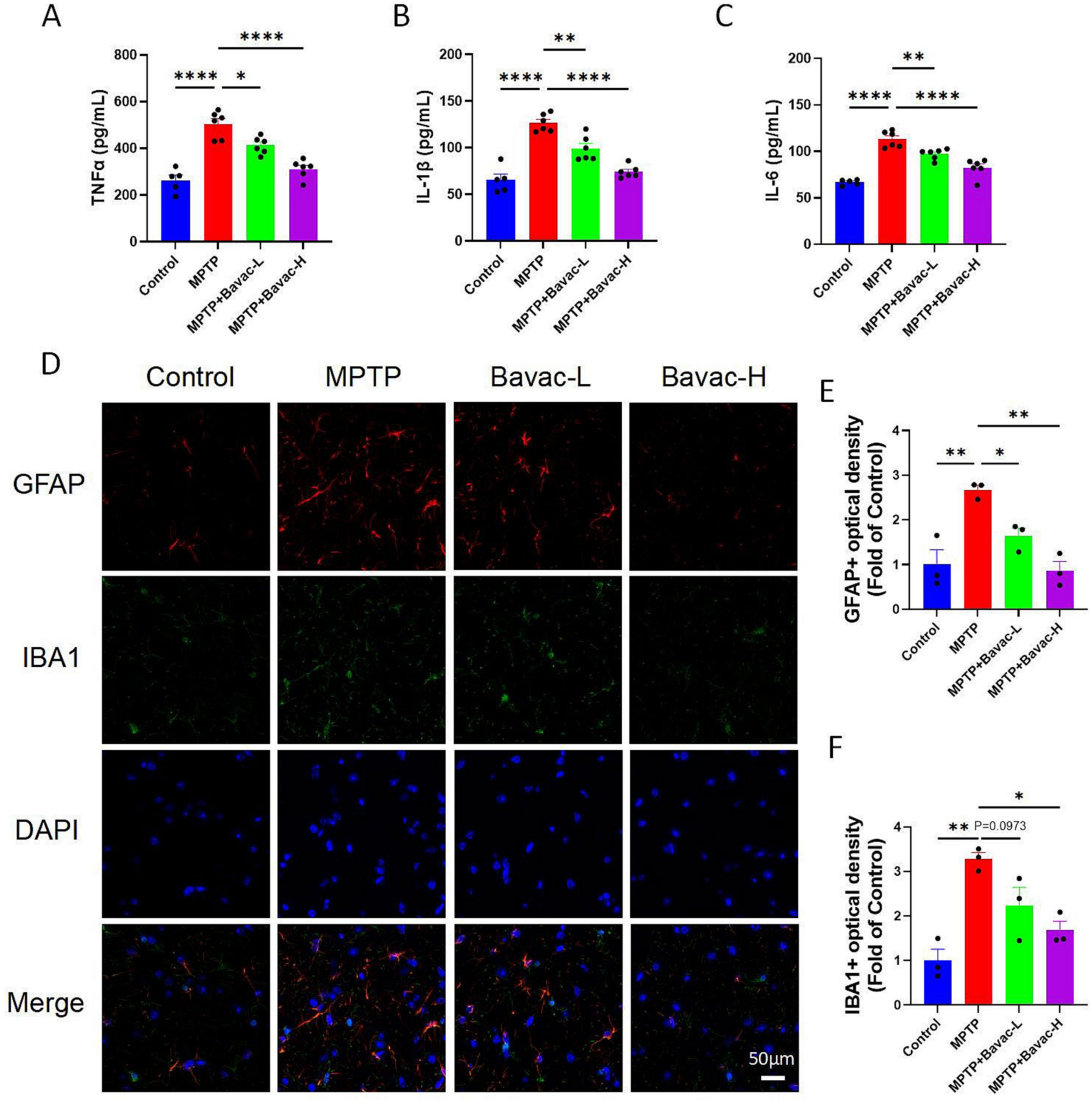
Bavachalcone reduces systemic inflammation and glial activation in MPTP-induced PD mice. **(A–C)** ELISA analysis of serum levels of proinflammatory cytokines TNF-*α*, IL-1β, and IL-6. MPTP administration significantly increased cytokine levels, which were attenuated by bavachalcone treatment in both low and high doses. n = 5–6 per group. **(D)** Representative immunofluorescence images of GFAP (astrocyte marker, red), IBA1 (microglial marker, green), and DAPI (nuclear stain, blue) in the substantia nigra. MPTP induced pronounced glial activation, whereas bavachalcone treatment mitigated astrocytic and microglial reactivity. Scale bar = 50 μm. **(E–F)** Quantification of GFAP and IBA1 immunofluorescence intensity in the substantia nigra (SN) of mice. *n* = 3 per group. Data are expressed as mean ± SEM. *p < 0.05, **p < 0.01, ***p < 0.001, ****p < 0.0001. PD, Parkinson’s disease; ELISA, enzyme-linked immunosorbent assay; SEM, standard error of the mean.

To determine whether bavachalcone alleviates inflammation in mice with PD, serum levels of proinflammatory cytokines were measured using ELISA. MPTP-treated mice exhibited significantly elevated levels of TNF-*α*, IL-1β, and IL-6 compared with those in the control group. Notably, bavachalcone treatment significantly reduced the levels of these cytokines in both low- and high-dose groups (Figures 3A–C).

To assess glial activation in the substantia nigra, immunofluorescence staining was performed to detect GFAP and IBA1 expression (Figures 3D–F). MPTP administration substantially increased GFAP and IBA1 expression, indicating significant activation of astrocytes and microglia, respectively. Bavachalcone effectively attenuated glial activation in a dose-dependent manner. These results indicate that bavachalcone mitigates both central and peripheral inflammatory responses in the MPTP-induced PD model.

Collectively, these findings demonstrate that bavachalcone exerts potent anti-inflammatory effects by suppressing systemic cytokine production and central glial activation in mice with PD.

### Bavachalcone modulates gut microbiota diversity in mice with MPTP-induced PD

Recent studies have indicated that dysbiosis of the gut microbiota plays a critical role in the pathogenesis of PD. To investigate whether bavachalcone improves Parkinsonian phenotypes by modulating the gut microbiota, we performed 16S rDNA sequencing of fecal samples from Control, MPTP, and bavachalcone-treated mice.

**Figure 4:**
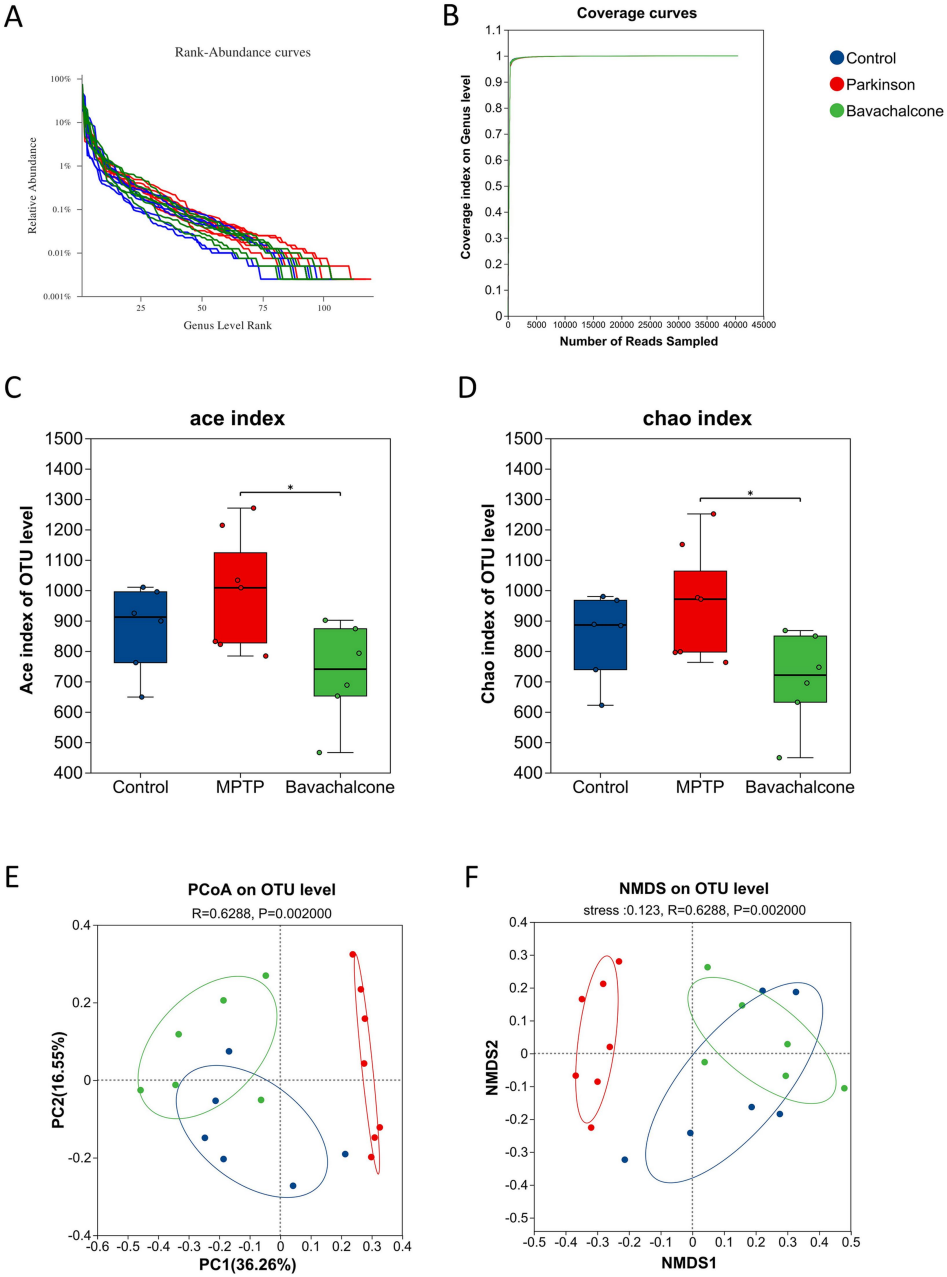
Bavachalcone modulates gut microbiota diversity in MPTP-induced PD mice. **(A)** Rank-abundance curves at the genus level across treatment groups. **(B)** Shannon diversity index. **(C)** Alpha diversity determined by ACE analysis. Kruskal–Wallis H test, *p < 0.05. **(D)** Alpha diversity based on Chao analysis. Kruskal–Wallis H test, *p < 0.05. **(E)** Principal Coordinate Analysis (PCoA) at the OTU level showing microbial community differences between groups. **(F)** NMDS analysis at the OTU level confirming community structure distinctions. PD, Parkinson’s disease; PCoA, Principal Coordinate Analysis; OTU, operational taxonomic unit; NMDS, Non-metric Multidimensional Scaling.

The rank-abundance curve reflected high species richness and even distribution within the microbial community (Figure 4A). A curve was plotted with the amount of extracted data on the x-axis and alpha diversity index on the y-axis. Flattening of the rarefaction curves indicated that the sequencing depth was sufficient (Figure 4B). *α*-Diversity analysis revealed significant differences in gut microbiota richness among the control group, MPTP-treated group, and bavachalcone-intervention group (Figures 4C,D). In *β*-diversity analysis, both principal coordinate analysis and non-metric multidimensional scaling revealed distinct differences in gut microbiota composition among the treatment groups (Figures 4E,F).

Collectively, these results indicate that bavachalcone effectively modulates the diversity and composition of gut microbiota disrupted by MPTP treatment. Bavachalcone may help alleviate gut dysbiosis associated with PD, offering new insights into its potential therapeutic mechanisms.

### Bavachalcone reshapes gut microbiota composition in mice with PD

**Figure 5:**
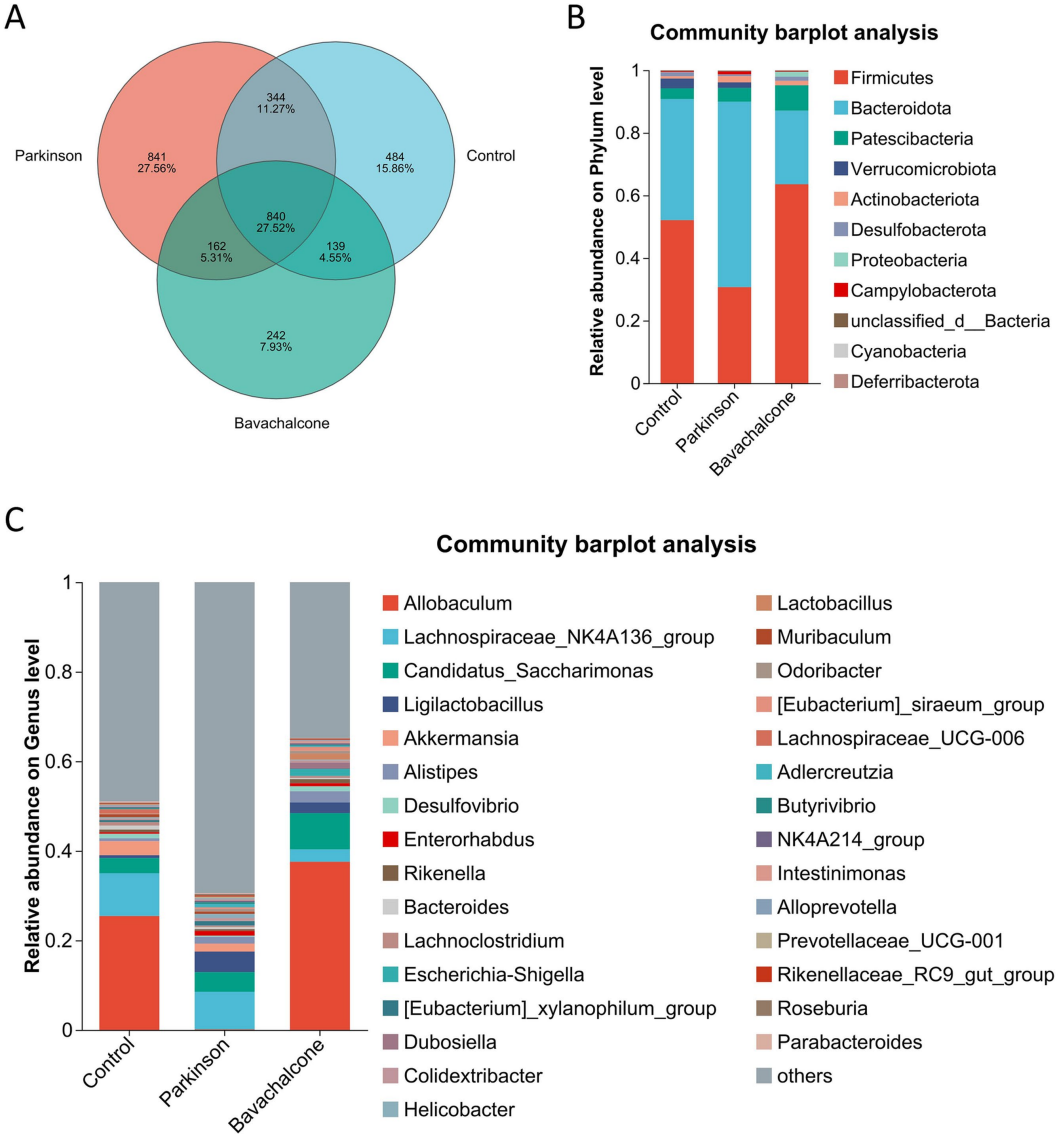
Bavachalcone reshapes gut microbiota composition at different taxonomic levels in PD mice. **(A)** Venn diagram showing shared and unique OTUs among treatment groups. **(B)** Community bar plot analysis at the phylum level. **(C)** Community bar plot analysis at the genus level. PD, Parkinson’s disease; OTU, operational taxonomic unit.

The Venn diagram demonstrated partial overlap in operational taxonomic units among the three groups, with the bavachalcone group displaying a unique microbial profile, suggesting its potential for targeted microbial modulation (Figure 5A).

To further explore the specific effects of bavachalcone on gut microbiota composition, we generated community bar plots at different taxonomic levels, including phylum (Figure 5B) and genus (Figure 5C). At the phylum level, Firmicutes were enriched in the Control and bavachalcone groups but markedly decreased in the MPTP group. By contrast, the relative abundance of Bacteroidetes, Actinobacteria, and Campylobacterota was elevated in the MPTP group, indicating dysbiosis, which was partially reversed by bavachalcone treatment (Figure 5B). At the genus level, *Allobaculum* was highly enriched in both the Control and bavachalcone groups, whereas its abundance was significantly reduced in the MPTP group. By contrast, *Ligilactobacillus* was significantly elevated in the MPTP group, and this increase was suppressed by bavachalcone (Figure 5C). These results suggest that bavachalcone alleviates gut dysbiosis by reducing the abundance of harmful genera and restoring beneficial taxa.

**Figure 6:**
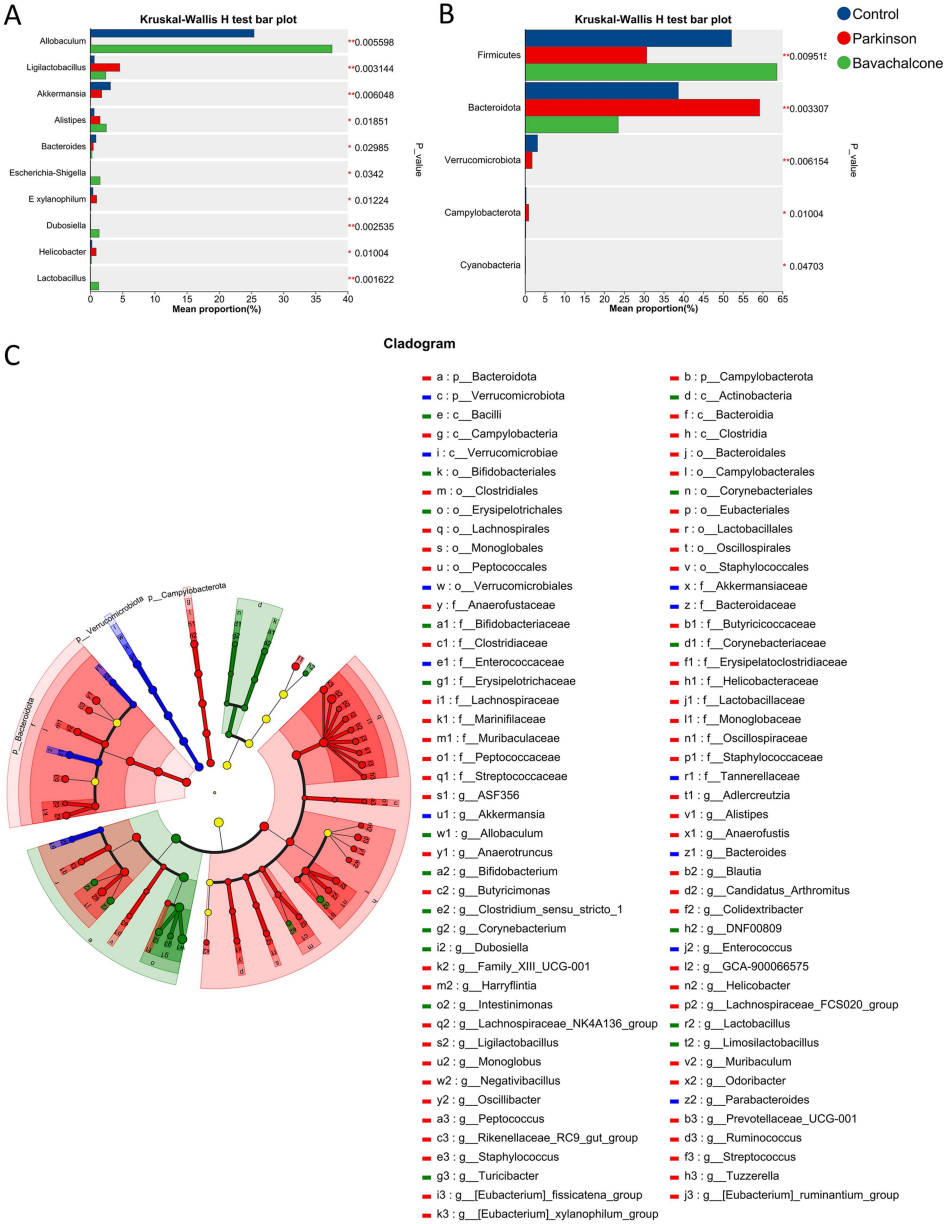
Differential gut microbiota analysis. **(A)** Kruskal–Wallis H test for differential genera. **(B)** Kruskal–Wallis H test for differential phyla. **(C)** Cladogram generated by LEfSe showing taxonomic biomarkers across treatment groups.

To identify key differential taxa influenced by bavachalcone, we performed intergroup differential significance testing. At the genus level, *Allobaculum* was significantly enriched in the Control and bavachalcone groups but markedly reduced in the MPTP group. By contrast, *Ligilactobacillus*, *Eubacterium xylanophilum*, and *Helicobacter* were significantly enriched in the MPTP group and reduced in the Control and bavachalcone groups (Figure 6A). At the phylum level, Firmicutes were enriched in the Control and bavachalcone groups but significantly decreased in the MPTP group. Conversely, Bacteroidetes, Campylobacteroides, and Cyanobacteria were elevated in the MPTP group and reduced in the Control and bavachalcone groups (Figure 6B). Furthermore, the cladogram generated from phylogenetic analysis illustrated the evolutionary relations among differential taxa, suggesting that bavachalcone may exert neuroprotective effects through the modulation of specific microbial pathways (Figure 6C).

### Bavachalcone regulates serum metabolites in mice with MPTP-induced PD

**Figure 7:**
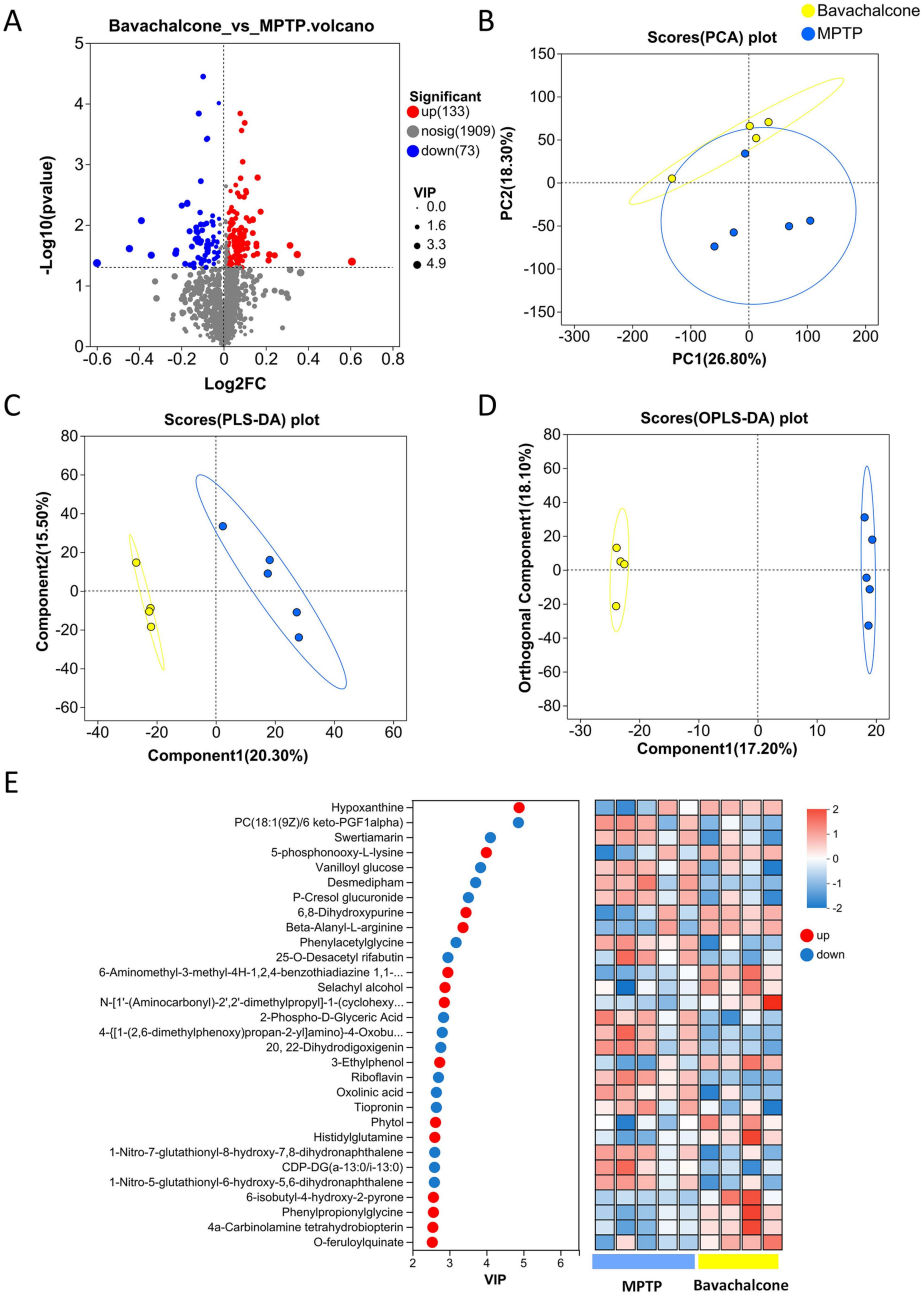
Bavachalcone alters serum metabolic profiles in MPTP-induced PD mice. **(A)** Volcano plot of differentially abundant metabolites between bavachalcone-treated and MPTP groups. **(B)** PCA plot of serum metabolites. **(C)** PLS-DA score plot. **(D)** OPLS-DA score plot. **(E)** VIP plot ranking key metabolites from the PLS-DA model and heatmap of differential metabolite expression. PD, Parkinson’s disease; PCA, Principal Component Analysis; PLS-DA, Partial Least Squares Discriminant Analysis; OPLS-DA, Orthogonal Partial Least Squares Discriminant Analysis; VIP, variable importance in projection.

To investigate the effects of bavachalcone on systemic metabolic profiles in mice with MPTP-induced PD, serum metabolomic analysis was performed. The volcano plot showed a number of significantly altered metabolites between the bavachalcone-treated and MPTP groups, including 133 upregulated and 73 downregulated metabolites (Figure 7A). Principal component analysis demonstrated distinct clustering between the two groups, indicating treatment-related metabolic shifts (Figure 7B). Furthermore, supervised PLS-DA and orthogonal PLS-DA confirmed a clear separation between groups, suggesting that bavachalcone treatment substantially altered serum metabolite composition (Figures 7C,D).

To further identify the key metabolites contributing to group-level metabolic differences, variable importance in projection analysis based on the PLS-DA model was performed (Figure 7E). Metabolites, such as hypoxanthine, swertiamarin, riboflavin, and tiopronin, were identified as major contributors to intergroup separation. Additionally, a heatmap of the top-ranking metabolites revealed distinct expression patterns between the MPTP and bavachalcone-treated groups, with bavachalcone partially restoring the levels of several disrupted metabolites. These findings suggest that bavachalcone exerts significant regulatory effects on the serum metabolic profile, which may underlie its neuroprotective action in PD.

**Figure 8:**
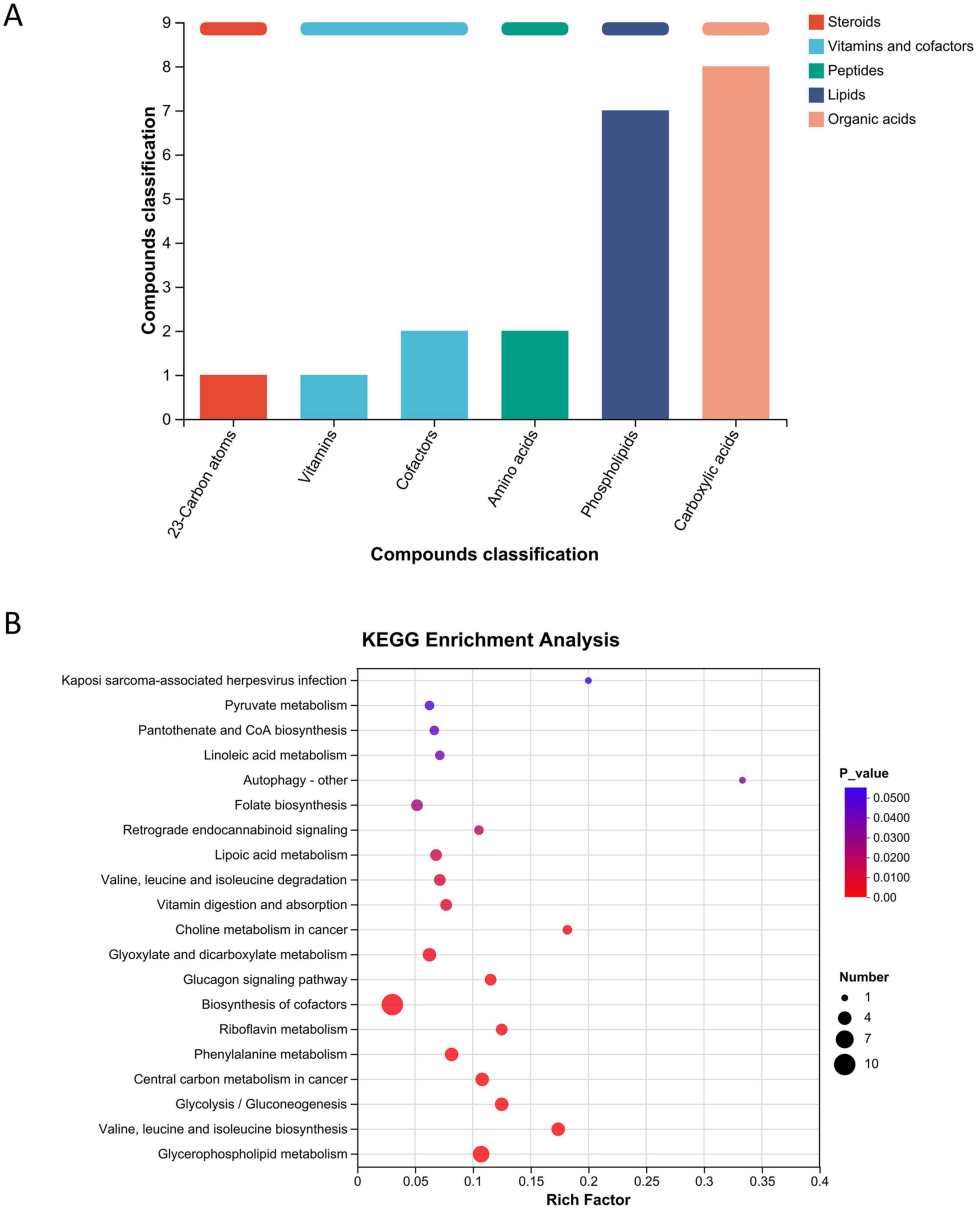
Classification and pathway enrichment analysis of differential metabolites. **(A)** Compound classification of differentially abundant metabolites. **(B)** KEGG pathway enrichment analysis comparing bavachalcone-treated and MPTP groups.

Further classification of the differentially abundant metabolites indicated that most belonged to compound classes, such as carboxylic acids, phospholipids, amino acids, and vitamins (Figure 8A). KEGG pathway enrichment analysis revealed that the altered metabolites were primarily associated with pyruvate metabolism, folate biosynthesis, glycolysis/gluconeogenesis, and phenylalanine metabolism—pathways closely related to the pathogenesis of PD (Figure 8B).

These results suggest that bavachalcone may exert its neuroprotective effects in PD, at least in part, by remodeling systemic metabolism and restoring key metabolic pathways disrupted by MPTP treatment.

## Discussion

This study provides comprehensive evidence that bavachalcone, a prenylated chalcone derived from *Psoralea corylifolia*, exerts neuroprotective effects in an MPTP-induced murine model of PD. Our findings revealed that bavachalcone ameliorated motor dysfunction, preserved dopaminergic neurons in the nigrostriatal pathway, and modulated both gut microbiota composition and serum metabolic profiles. These results highlight the therapeutic potential of bavachalcone in PD through its multitarget actions on the gut–brain axis and systemic metabolic regulation.

In our study, bavachalcone administration significantly ameliorated motor deficits in MPTP-induced Parkinson’s model mice, as evidenced by improved performance in the pole, rotarod, and open field tests. These behavioral improvements were accompanied by preservation of TH-positive neurons in the substantia nigra and striatum, indicating protection of dopaminergic neurons. The neuroprotective effects of bavachalcone are likely attributable to its antioxidant properties, as chalcone derivatives have been reported to activate the Nrf2/ARE pathway, thereby reducing oxidative stress—a key contributor to PD pathogenesis (Dang et al., 2015; Wu et al., 2023). Furthermore, chalcones have been shown to exert anti-inflammatory effects by inhibiting microglial activation and suppressing proinflammatory mediators, such as COX-2 and iNOS, which may further contribute to the observed neuroprotection (Parambi et al., 2020).

In addition to gut microbiota dysbiosis, systemic and central inflammation are increasingly recognized as key contributors to the pathophysiology of PD. Elevated levels of proinflammatory cytokines, such as TNF-*α*, IL-1β, and IL-6, have been consistently reported in the serum and cerebrospinal fluid of patients with PD and are known to correlate with disease severity and progression (Chen et al., 2018; Qin et al., 2016; Tansey et al., 2022). In the present study, MPTP administration significantly increased the serum levels of these cytokines, indicating a robust peripheral inflammatory response. Notably, bavachalcone treatment markedly reduced cytokine expression in a dose-dependent manner, suggesting potent systemic anti-inflammatory activity.

Moreover, immunofluorescence analysis of the substantia nigra revealed pronounced activation of astrocytes and microglia following MPTP exposure, as indicated by increased expression of GFAP and IBA1, respectively. Glial activation, a hallmark of neuroinflammation, has been implicated in dopaminergic neuronal loss (Isik et al., 2023; Kam et al., 2020). Meanwhile, recent evidence has demonstrated that the gut microbiome is essential for movement disorders, microglial activation, and α-synuclein pathology (Sampson et al., 2016). Bavachalcone significantly suppressed glial reactivity, indicating its capacity to attenuate neuroinflammatory processes in the central nervous system. Together, these findings suggest that bavachalcone may exert neuroprotective effects not only by modulating the gut microbiota but also by mitigating both systemic and central inflammatory responses, thereby offering a multifaceted mechanism of action in PD.

Recent research has highlighted the crucial role of the gut microbiota in the pathogenesis and progression of PD, suggesting that microbiome-targeted therapies may provide promising therapeutic avenues. In this study, we demonstrated that bavachalcone significantly modulated gut microbial composition in MPTP-induced Parkinson’s model mice, as evidenced by distinct microbial profiles and partially restored community structures at both the phylum and genus levels.

Our results showed that the gut microbiota in the MPTP group exhibited typical dysbiotic features, including a decrease in Firmicutes and an increase in Bacteroidota, consistent with prior reports (Jiao et al., 2025) linking microbial imbalance to PD-related neuroinflammation and intestinal barrier dysfunction (Hu et al., 2024; Jiao et al., 2025). Cyanobacteria were enriched in the MPTP group, and several cyanobacterial toxins are known to act through multiple molecular mechanisms and exhibit high neurotoxicity (Sini et al., 2021). Bavachalcone treatment partially reversed these changes, suggesting a rebalancing effect on the gut microbial ecosystem. The abundance of short-chain fatty acid (SCFA)-producing bacteria is significantly reduced in the intestines of patients with PD, and SCFAs play an important role in maintaining intestinal barrier function and regulating neuroinflammation (Kalyanaraman et al., 2024). At the genus level, bavachalcone significantly restored the abundance of *Allobaculum*, a known SCFA-producing genus that supports gut barrier integrity and possesses anti-inflammatory properties (Li et al., 2023; Wang et al., 2018; Xu et al., 2024; Zhou et al., 2024). Additionally, bavachalcone suppressed the overgrowth of potentially pathogenic taxa, such as *Ligilactobacillus*, *Escherichia*–*Shigella*, and *Helicobacter*, which have been associated with gastrointestinal inflammation and may exacerbate Parkinsonian symptoms (Gámez-Macías et al., 2024; Lehours and Ferrero, 2019; Q. Li et al., 2021).

Moreover, phylogenetic cladogram analysis revealed that these bavachalcone-responsive taxa were phylogenetically distinct, indicating a broad-spectrum impact on microbial networks. The restoration of beneficial taxa and suppression of harmful genera suggest that bavachalcone may exert neuroprotective effects via the gut–brain axis, potentially by modulating microbial metabolites, immune signaling, or intestinal permeability. In summary, these findings indicate that bavachalcone alleviates PD–associated gut dysbiosis by reshaping microbial community structure, enriching beneficial bacteria, and suppressing harmful taxa. This microbial reprogramming may represent one of the mechanisms contributing to the therapeutic effects of bavachalcone in PD and warrants further mechanistic investigation.

Our study demonstrated that bavachalcone significantly altered the diversity and abundance of serum differential metabolites in PD mice, with notably elevated levels of hypoxanthine, 5-phosphonooxy-L-lysine, 6,8-dihydroxypurine, and Beta-alanyl-L-arginine, while markedly reduced levels of swertiamarin, vanilloyl glucose, desmedipham, and phenylacetylglycine were observed; enrichment analysis of these differential metabolites indicates that bavachalcone normalized MPTP-induced disruptions in pyruvate metabolism, folate biosynthesis, and phenylalanine metabolism. Pyruvate metabolism is essential for mitochondrial energy production, and its dysregulation exacerbates oxidative stress in PD (Mallet et al., 2022; Quansah et al., 2018). The upregulation of riboflavin and hypoxanthine—metabolites with antioxidant and neurotrophic properties—suggests that bavachalcone enhances endogenous neuroprotection (Ebadi et al., 1998). Furthermore, the restoration of phenylalanine metabolism may mitigate dopamine depletion, as phenylalanine is a precursor of TH (Fernstrom and Fernstrom, 2007).

Although our study demonstrated the efficacy of bavachalcone in a preclinical model, several translational challenges remain. First, the precise molecular targets through which bavachalcone modulates the gut microbiota and metabolic pathways require validation using germ-free or antibiotic-treated models. Second, the dose-dependent effects observed (Bavac-H vs. Bavac-L) warrant pharmacokinetic studies to define optimal therapeutic windows. Third, we conducted experiments exclusively on C57BL/6 mice without extending to additional mouse strains such as CD-1 mice. Fourth, while we examined differential metabolite levels in mouse serum, we did not assess differential metabolites in the intestinal tract; nor did we investigate the functional consequences of these core differential metabolites on PD mice. Finally, clinical studies are necessary to confirm these findings, particularly in light of species-specific differences in gut microbiota composition and drug metabolism.

## Conclusion

Our study provides evidence that bavachalcone exerts protective effects against MPTP-induced PD phenotypes by improving motor function, preserving dopaminergic neurons, suppressing inflammation, modulating gut microbiota, and remodeling systemic metabolism. These findings support the potential of bavachalcone as a promising therapeutic candidate for PD.

## Data Availability

The raw sequencing data reported in this paper have been deposited in the Genome Sequence Archive in National Genomics Data Center, China National Center for Bioinformation / Beijing Institute of Genomics, Chinese Academy of Sciences (GSA: CRA029771) that are publicly accessible at https://ngdc.cncb.ac.cn/gsa.
